# Mild and Severe SARS-CoV-2 Infection Induces Respiratory and Intestinal Microbiome Changes in the K18-hACE2 Transgenic Mouse Model

**DOI:** 10.1128/spectrum.00536-21

**Published:** 2021-08-11

**Authors:** Brittany Seibert, C. Joaquín Cáceres, Stivalis Cardenas-Garcia, Silvia Carnaccini, Ginger Geiger, Daniela S. Rajao, Elizabeth Ottesen, Daniel R. Perez

**Affiliations:** a Department of Population Health, College of Veterinary Medicine, University of Georgia, Athens, Georgia, USA; b Tifton Diagnostic Laboratory, College of Veterinary Medicine, University of Georgia, Tifton, Georgia, USA; c Department of Microbiology, University of Georgia, Athens, Georgia, USA; Fundacio irsiCaixa

**Keywords:** SARS-CoV-2, mouse, microbiome, COVID-19, lung, intestine, cecum, respiratory infection

## Abstract

Transmission of the severe acute respiratory syndrome coronavirus 2 (SARS-CoV-2) has resulted in millions of deaths and declining economies around the world. K18-hACE2 mice develop disease resembling severe SARS-CoV-2 infection in a virus dose-dependent manner. The relationship between SARS-CoV-2 and the intestinal or respiratory microbiome is not fully understood. In this context, we characterized the cecal and lung microbiomes of SARS-CoV-2-challenged K18-hACE2 transgenic mice in the presence or absence of treatment with the M^pro^ inhibitor GC-376. Cecum microbiome showed decreased Shannon and inverse (Inv) Simpson diversity indexes correlating with SARS-CoV-2 infection dosage and a difference of Bray-Curtis dissimilarity distances among control and infected mice. Bacterial phyla such as Firmicutes, particularly, Lachnospiraceae and Oscillospiraceae, were significantly less abundant, while Verrucomicrobia, particularly, the family Akkermansiaceae, were increasingly more prevalent during peak infection in mice challenged with a high virus dose. In contrast to the cecal microbiome, the lung microbiome showed similar microbial diversity among the control, low-, and high-dose challenge virus groups, independent of antiviral treatment. Bacterial phyla in the lungs such as Bacteroidetes decreased, while Firmicutes and Proteobacteria were significantly enriched in mice challenged with a high dose of SARS-CoV-2. In summary, we identified changes in the cecal and lung microbiomes of K18-hACE2 mice with severe clinical signs of SARS-CoV-2 infection.

**IMPORTANCE** The COVID-19 pandemic has resulted in millions of deaths. The host’s respiratory and intestinal microbiome can affect directly or indirectly the immune system during viral infections. We characterized the cecal and lung microbiomes in a relevant mouse model challenged with a low or high dose of severe acute respiratory syndrome coronavirus 2 (SARS-CoV-2) in the presence or absence of an antiviral M^pro^ inhibitor, GC-376. Decreased microbial diversity and taxonomic abundances of the phyla Firmicutes, particularly, Lachnospiraceae, correlating with infection dosage were observed in the cecum. In addition, microbes within the family Akkermansiaceae were increasingly more prevalent during peak infection, which is observed in other viral infections. The lung microbiome showed similar microbial diversity to that of the control, independent of antiviral treatment. Decreased Bacteroidetes and increased Firmicutes and Proteobacteria were observed in the lungs in a virus dose-dependent manner. These studies add to a better understanding of the complexities associated with the intestinal microbiome during respiratory infections.

## INTRODUCTION

Throughout 2020, the World Health Organization reported ∼8 million confirmed COVID-19 cases and ∼1.8 million confirmed deaths, leading to a continuous increase of cases during the early months of 2021 (1). The severe acute respiratory syndrome coronavirus 2 (SARS-CoV-2) virus replicates and migrates to multiple tissues, including the airways and alveolar epithelial cells in the lungs, triggering a strong immune response that may lead to exacerbation of inflammatory responses, a major complication in SARS-CoV-2 patients (2–9). While many infected patients can present as asymptomatic, others show clinical manifestations such as fever, shortness of breath, cough, headache, and occasional gastrointestinal symptoms (10–12); however, there are still several aspects of the host immune response that need to be elucidated.

The respiratory and intestinal microbiome can have direct impacts on host cells or an indirect impact on the immune system during viral infections (13, 14). Our knowledge of the microbiota’s role in essential physiological processes and disease progression has expanded greatly due to advanced sequencing technology (15) but remains poorly parameterized for many diseases. Previous studies have shown that the residential bacterial communities that reside in the respiratory tract can affect and/or be affected by respiratory viral infections, such as playing a role in the enhancement of influenza virus transmission by promoting environmental stability and infectivity (16, 17). Changes in the respiratory microbiome during influenza virus infection in mice showed decreased abundance of *Alphaproteobacteria* and increased abundances of *Gammaproteobacteria*, *Actinobacteria*, and facultative anaerobes such as Streptococcus and Staphylococcus (16). While the potential for respiratory diseases to impact the residential microbiome is clear, many studies have also observed impacts on the intestinal microbiome during respiratory infections (18–21). Reported changes in the intestinal microbiome include enrichment of Bacteroides and Proteobacteria along with a decrease in Firmicutes during respiratory viral infections such as influenza virus and respiratory syncytial virus (RSV) (18–21). Not only were changes observed in the intestinal bacterial communities, but it was demonstrated that TLR5 sensing of flagellated microbes in the intestine increased antibody responses post-influenza virus vaccination and that the oral administration of gut microbe Akkermansia muciniphila reduced weight loss and mortality during highly pathogenic influenza virus infection (22, 23). It has also been suggested that microbiome changes or “gut dysbiosis” can lead to gut permeability, resulting in secondary infections such as pneumococcal disease (20, 21). Therefore, we were interested in exploring the impacts of SARS-CoV-2 infection on the microbiome through the use of a mouse model.

The relationship between SARS-CoV-2 and the intestinal or respiratory environment, particularly, its impact on the microbiome, is not fully understood. Previous studies have looked at SARS-CoV-2-induced changes in the nasopharynx and fecal microbiome of humans and have found a general decrease of bacterial diversity correlating with disease severity (5, 24–28). Microbiome diversity and composition differences were not observed in the nasopharynx of negative and positive PCR patients in one study analyzing patients with mild disease (28). However, another study found a decrease in the nasopharyngeal microbial diversity, and that differences were linked to disease severity (27). Impacts on the fecal microbiome are also expected, as numerous studies have observed viral RNA in the feces of infected individuals (9, 29), and gastrointestinal upset during COVID-19 infection was reported occasionally (29). Overall, fecal microbiome studies have found a decrease in the gut microbiota diversity and abundance in SARS-CoV-2 patients compared to that in negative patients (5, 24, 26). Multiple bacterial genera that are associated with opportunistic pathogens such as Streptococcus, *Rothia*, *Veillonella*, *Erysipelatoclostridium*, *Actinomyces*, *Collinsella*, and *Morganella* had increased relative abundance in fecal samples collected from SARS-CoV-2 patients compared to that in the controls (5, 24). Furthermore, a recent study showed that the addition of oral bacteriotherapy treatment in human patients with SARS-CoV-2 displayed decreased mortality and reduced intensive care unit (ICU) hospitalizations (30). This suggests that understanding the host microbial changes during SARS-CoV-2 infection could help provide future treatment methods to overcome severe infections.

While nasopharynx and fecal samples can be informative, several studies in human and animal models suggest that the intestinal lumen and mucosa may be colonized by microbial communities that are different from rectal swabs or feces (31–33). However, deep respiratory and intestinal samples are more difficult to collect among human patients. Furthermore, the human microbiome is highly variable and impacted by diverse environmental conditions (34–37), which complicates analysis of human population studies. Therefore, analyzing the respiratory and intestinal microbiome of an animal model susceptible to SARS-CoV-2 in a controlled environment that mirrors mild or severe SARS-CoV-2 infection in humans would be beneficial in understanding the relationship between SARS-CoV-2 infection and the host microbiome.

Recent reports showed that K18-hACE2 mice develop disease resembling severe SARS-CoV-2 infection in a virus dose-dependent manner, mirroring partially what is observed in humans (38–45). We aimed to use this model to understand microbiome responses to SARS-CoV-2, particularly, infection or antiviral induced changes in the intestinal and lung microbiomes. The studies were performed in the context of mice challenged with two different doses of the SARS-CoV-2 virus and receiving either antiviral therapy with the M^pro^ inhibitor GC-376 or vehicle for 7 days post-virus challenge. We performed 16S sequencing at 2, 5, and 14 days postchallenge (dpc) with a prototypic SARS-CoV-2 strain. The results from the intestinal microbiome show microbial differences in alpha and beta diversity measures that are SARS-CoV-2 virus dose dependent and with little effect of GC-376 treatment on lung bacterial communities.

(Portions of this work were submitted to an online preprint archive [46].)

## RESULTS

### Clinical outcomes of K18-hACE2 transgenic mice challenged with two different doses of SARS-CoV-2 virus and samples for microbiome analyses.

**FIG 1:**
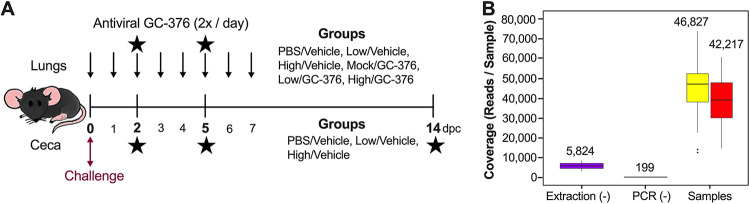
Study design and sample coverage across ceca and lung samples. (A) Study timeline for the mouse study. Six-week-old female mice were inoculated with PBS and low or high titers of SARS-CoV-2 virus. Three groups of mice were administered antiviral GC-376 twice per day starting 3 h after inoculation until 7 dpc (indicated by top arrows). Lung and cecum samples were collected at 2, 5, and 14 dpc (indicated by the stars). Lung samples were collected in vehicle and GC-376 groups, while cecum samples were collected from the vehicle group. (B) Sequencing coverage of the extraction blank, PCR blank, and samples (cecum, yellow; lung, red). Coverage mean is indicated above the boxplot. Outliers are indicated by points outside the plot. Three outliers for the ceca (>80,000) are not shown.

Taking advantage of a study evaluating antiviral activity of GC-376 against SARS-CoV-2 virus in the K18-hACE2 mouse model, we evaluated the microbiome composition at different times after SARS-CoV-2 challenge. We and others have shown that mice challenged with 10^3^ 50% tissue culture infectious dose (TCID_50_)/mouse of the SARS-CoV-2 virus (low/vehicle) presented with brief reduced activity and clinical signs leading to ∼60% survival (43). In contrast, mice challenged with 10^5^ TCID_50_/mouse of SARS-CoV-2 (high/vehicle) presented initially with relatively normal activity followed by rapid weight loss and substantial deterioration of clinical outcomes (43). By 6 dpc, mice in the high-virus-dose group showed ∼20% weight loss, and all mice died or had to be euthanized by 8 dpc (43). Peak virus titers for the low- and high-dose groups were observed at 2 and then 5 dpc in the nasal turbinates and lungs (43). Antiviral GC-376 treatment resulted in milder inflammation and reduced lesions and viral loads compared to those in the vehicle group, although it did not improve clinical outcomes (43). We analyzed the changes in the intestinal and respiratory microbiomes by collecting ceca and lungs from mice of the following groups: phosphate-buffered saline (PBS)/vehicle, low/vehicle, and high/vehicle (Fig. 1A). Because the respiratory tract is the primary site of replication for SARS-CoV-2, we also collected lung samples from the antiviral GC-376-treated groups (mock/GC-376, low/GC-376, and high/GC-376) to evaluate whether antiviral intervention would affect the residential respiratory microbiome (Fig. 1A).

We performed 16S sequencing at 2, 5, and 14 dpc except for lung samples in the PBS/vehicle group due to limited DNA concentrations. Of the total ceca and lungs combined, 5,098,781 raw reads were obtained, while 3,103,597 reads remained after DADA2 trimming, filtering, merging, and chimera removal. Two lung samples, one from the low/vehicle group at 2 dpc and one from the high/vehicle group at 5 dpc were removed from the analysis because of low coverage (<10,000 reads). One lung sample from the mock/GC-376 group at 14 dpc, considered an outlier according to Grubbs test on taxonomic abundance (*P* = 6.022e−07), was also removed from the analysis. Because the microbiome of the lungs can be easily contaminated, we compared the sequencing coverage of the blank extractions and negative PCR controls to the samples from the lungs and ceca (Fig. 1B). Blank extraction samples had an average of 5,824 reads/sample, and the negative PCR controls had an average of 199 reads/sample. Meanwhile, cecum samples had an average of 46,827 with a minimum of 12,892 reads/sample, and the lung samples had an average of 42,217 with a minimum of 14,808 reads/sample (Fig. 1B). Since the number of reads for the ceca and lungs are notably greater than those for the blanks, the difference in coverage suggests that the majority of the reads in the samples are not from cross-contamination.

### Microbial diversity in the cecum of SARS-CoV-2 challenge K18-hACE2 mice.

**FIG 2:**
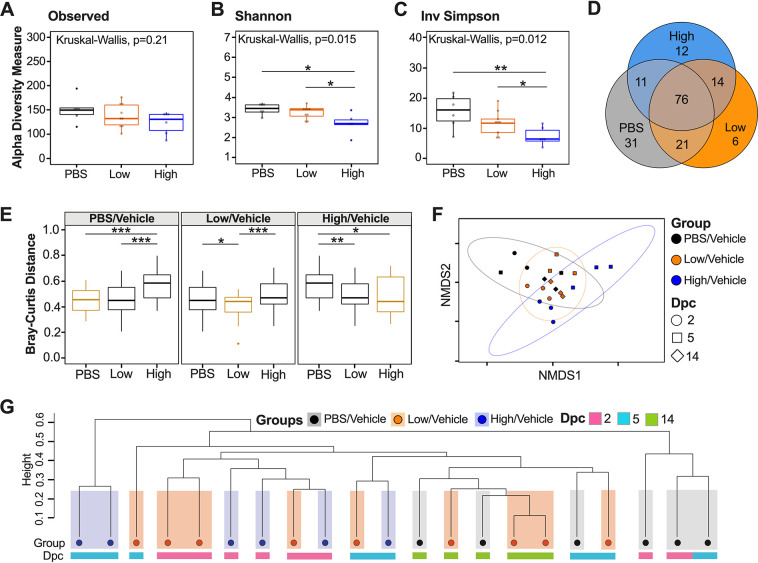
Alpha and beta diversity metrics of cecum samples. Comparison of observed ASVs (A), Shannon diversity index (B) and Inv Simpson (C) of different groups (PBS/vehicle, black; low/vehicle, orange; high/vehicle, blue) containing all time points (dpc) from rarified ASV count table. (D) Venn diagram of rarified ASV counts comparing the three different groups. (E) Comparison of weighted Bray-Curtis dissimilarity distances within each group and across different groups. Gold boxes represent within-group variation, while black boxes represent the between-group variation. (F) NMDS plot of weighted Bray-Curtis dissimilarity distance. Days postchallenge are indicated by the shape, and groups are indicated by color. Ellipses were constructed using a multivariate *t* distribution. (G) Dendrogram showing the relationship of different groups and time points (dpc) using Bray-Curtis dissimilarity distance. Hierarchical cluster analysis was performed using hclust with agglomeration method average. Shaded colors and circles correspond to the different groups as described previously. Colored bars below the circles represent the different time points (pink, 2 dpc; light blue, 5 dpc; green, 14 dpc). All statistical tests were performed using Kruskal-Wallis or Wilcox-rank tests for pairwise comparisons. *, *P* < 0.05; **, *P* < 0.005; ***, *P* < 0.0005.

To evaluate spatial differences in microbial diversity and community structure in the cecum, we evaluated alpha diversity using count data from rarified amplicon sequence variants (ASVs) to calculate the number of observed variants and the Shannon and inverse (Inv) Simpson indexes. There were no significant differences in ASV richness among PBS/vehicle, low/vehicle, and high/vehicle groups when samples from each time point (dpc) were combined (Fig. 2A). Analyses of each group at each time point showed a trend toward increased number of ASVs as the number of days postchallenge increased for all groups; however, statistical testing was not performed because of the limited sample size per time point (*n* = 2 or 3) (see Fig. S1A in the supplemental material). Shannon and Inv Simpson indexes varied significantly between groups (Kruskal-Wallis *P* = 0.015 and *P* = 0.012, respectively) (Fig. 2B and C). The PBS/vehicle group had the highest Shannon and Inv Simpson indexes, followed by low/vehicle and then high/vehicle groups (Fig. 2B and C). Pairwise comparisons showed that PBS/vehicle and low/vehicle groups had significantly higher Shannon and Inv Simpson diversity indexes than the high/vehicle group (Wilcox-rank test *P* = 0.015, *P* = 0.012 and *P* = 0.0087, *P* = 0.02, respectively) (Fig. 2B and C). Shannon diversity and Inv Simpson indexes of each group at each time point showed a similar trend among days after challenge (Fig. S1B and C). In particular, the largest difference among the PBS/vehicle and low/vehicle groups compared to the high/vehicle group was at 5 dpc, which correlates with the greatest body weight change for the high/vehicle group (Fig. S1B and C). However, there was no statistical difference among 2-, 5-, and 14-dpc time points when analyzing the virus challenge groups (low/vehicle and high/vehicle) (see Fig. S2). Taken together, the alpha diversity indexes showed that the microbial diversity in the ceca of K18-hACE2 mice correlates inversely with SARS-CoV-2 virus challenge dose.

To assess the relationship between microbial community structure and SARS-CoV-2 challenge during the course of infection, we analyzed the number of shared ASVs. The three groups shared 76 ASVs after the count data were rarified with a detection limit of 0.001 in at least 90% of the samples (Fig. 2D). Using the same criteria, the PBS/vehicle group had 31 unique ASVs, while the low/vehicle and high/vehicle groups had 6 and 12 unique ASVs, respectively (Fig. 2D). The low/vehicle group shared 21 ASVs with the PBS/vehicle group and 11 ASVs with the high/vehicle group, suggesting a more similar microbial composition between low/vehicle and PBS/vehicle groups (Fig. 2D). Next, we quantified changes of the cecum microbiome composition among different SARS-CoV-2-infected groups by comparing weighted dissimilarity distances (Bray-Curtis) within and across groups (Fig. 2E). Overall, the high/vehicle group showed greater dissimilarity to the PBS/vehicle group than to the low/vehicle group in all group comparisons (Fig. 2E). A nonmetric multidimensional scaling (NMDS) plot of the Bray-Curtis dissimilarity distance was used to assess the relationship between microbial community structure and SARS-CoV-2 challenge during the course of infection. The NMDS showed a spread of samples; however, the PBS/vehicle group showed more overlap with the low/vehicle group than with the high/vehicle group, which was further supported by a permutational multivariate analysis of variance (PERMANOVA) analyzing difference of groups (*P* = 0.001) (Fig. 2F). We further investigated the relationship of the three groups across days postchallenge by producing a hierarchical cluster analysis using the Bray-Curtis dissimilarity distances (Fig. 2G). While the samples did not cluster exclusively by treatment, in all cases, the high/vehicle group clustered with samples within their group or low/vehicle treatment samples to the exclusion of the PBS/vehicle group, and similarly, the PBS/vehicle samples clustered with their own group or the low/vehicle group to the exclusion of the high/vehicle group (Fig. 2G). Collectively, the beta diversity metrics suggest that a higher dosage of SARS-CoV-2 virus infection has a larger effect on microbial diversity and community structure than a low-virus-dose infection.

**FIG 3:**
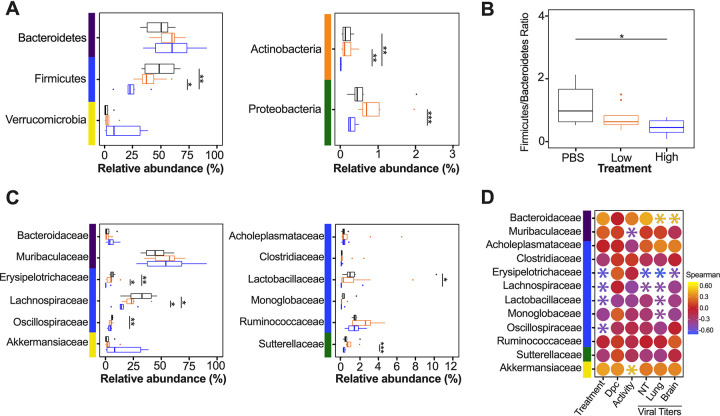
Relative abundance (%) of microbial communities in the ceca at the phylum and family levels. (A) Relative abundances (%) of the most predominant phyla were compared via box plots for each group (PBS/vehicle, black; low/vehicle, orange; high/vehicle, blue). Each box represents the interquartile range (first and third quartiles) of taxon abundance, and the line corresponds to the median abundance. Vertical lines represent variation in abundance, and the circles represent outliers. Corresponding phyla are noted by the colored bars on the left (purple, Bacteroidetes; blue, Firmicutes; yellow, Verrucomicrobia; orange, Actinobacteria; green, Proteobacteria). (B) Firmicutes/Bacteroidetes ratios were calculated and graphed to analyze differences among different groups. (C) Relative abundances (%) of the most abundant families were compared via box plots. Corresponding phyla are noted by the colored bar on left and in accordance with those in panel A. (D) Spearman correlations by family abundance. Corresponding phyla are noted by the colored bar to the on the left and in accordance with those in panel A. Significant values (*P* < 0.05) are demonstrated as an asterisk, while nonsignificant correlations are as a circle. Correlative factors include treatment (administered PBS, a low dose, or high dose), days postchallenge (2, 5, or 14), activity score (response to the environment and personnel stimulation; higher score indicates decreased activity), or viral titers measured in the nasal turbinates (NT), lung, and brain. Data (activity score, viral titers in the NT, lung, and brain) were adapted from Cáceres et al. (43). All statistical tests were performed using the Wilcox-rank test. *, *P* < 0.05; **, *P* < 0.005; ***, *P* < 0.0005.

**FIG 4:**
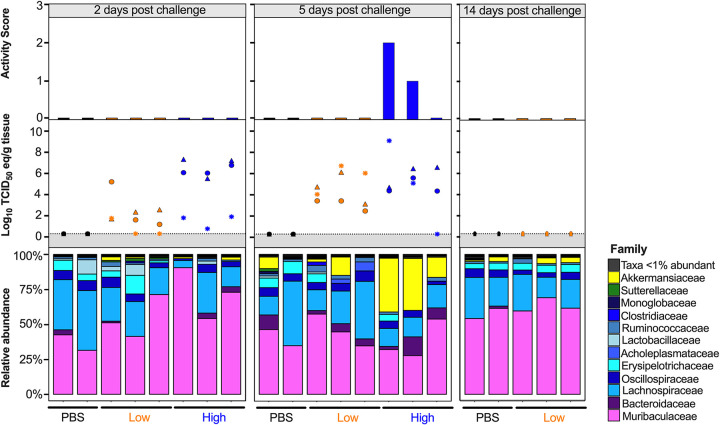
Relative abundance (%) of microbial communities in the ceca at the family level. Relative abundances (%) of individuals were calculated by agglomerating at the family level and then transformed into relative abundances. Taxa that had less than 1% abundance were grouped together. Groups are indicated by the bars at the bottom of the graph and color (PBS/vehicle, black; low/vehicle, orange; high/vehicle, blue). Each set of graphs is separated by days postchallenge indicated at the top. Viral nasal turbinates (circle), lung (triangle), and brain (asterisk) titers of individual mice that underwent microbial analysis are shown in the box above the microbial taxonomic abundances. Activity scores (response to the environment and personnel stimulation; higher score indicates decreased activity) for each mouse are shown above the viral titers. Data including activity score, viral titers in the NT, lung and brain were adapted from Cáceres et al. (43).

Since the diversity metrics suggested a difference among the low- and high-virus-dose-infected mice, we investigated those differences further by analyzing the relative abundance of the microbial communities at the phylum and family levels. The most prominent phyla were Bacteroidetes, Firmicutes, Verrucomicrobia, Actinobacteria, and Proteobacteria (Fig. 3A). The high/vehicle group had significantly lower relative abundance of Firmicutes and Actinobacteria than the PBS/vehicle and low/vehicle groups (Wilcox-rank test *P* = 0.0087, *P* = 0.02 and *P* = 0.005 and *P* = 0.002, respectively) (Fig. 3A). The low/vehicle group had significantly more abundance of Proteobacteria than the high/vehicle group (Wilcox-rank test *P* = 0.0008) (Fig. 3A). While not statistically significant, the high/vehicle group had notably higher abundance of Verrucomicrobia than the other two groups. Next, we looked at the relationship between the two predominant phyla to calculate the Firmicutes/Bacteroidetes (F/B) ratio. The PBS/vehicle group had the highest F/B ratio, followed by low/vehicle and then high/vehicle group (Fig. 3B). Pairwise comparisons showed that PBS/vehicle group had a significantly higher F/B ratio than the high/vehicle group (Wilcox-rank test *P* = 0.02) (Fig. 3B). When analyzing taxonomic diversity at the family level, Bacteroidaceae and Muribaculaceae were similar among groups when all sample time points were combined, consistent with its parent phylum, Bacteroidetes (Fig. 3C). The PBS/vehicle and low/vehicle groups had significantly higher abundances of Erysipelotrichaceae and Lachnospiraceae than the high/vehicle group (Wilcox-rank test *P* = 0.008, *P* = 0.02 and *P* = 0.05, *P* = 0.041, respectively) (Fig. 3C). Additional families in the phyla Firmicutes, Oscillospiraceae, and Lactobacillaceae had significantly increased abundances in the PBS/vehicle group compared to those in the high/vehicle group (Wilcox-rank test *P* = 0.0087 and *P* = 0.03, respectively) (Fig. 3C). While not significant, the family driving the increase shift of Verrucomicrobia in the high/vehicle group was Akkermansiaceae (Fig. 3C). While performing a correlation analysis among bacterial families and viral infection factors, Bacteroidetes families, including Bacteroidaceae, positively correlated with increased viral titers in the lung and brain, while Muribaculaceae was negatively correlated to activity score (Fig. 3D). Meanwhile, families of the phylum Firmicutes were negatively correlated with infection factors, including treatment and viral titers in the nasal turbinates, lung, and brain (Fig. 3D). On the other hand, Akkermansiaceae was positively correlated with increased activity score (Fig. 3D). This family was notably enriched at 5 dpc in 2 of 3 samples of the high/vehicle group (Fig. 4). Taken together, the taxonomic relative abundances displayed distinct changes at the phylum and family levels for the high-dose-infected group, while the control and low-dose groups were more similar, particularly in families within the phyla Bacteroidetes, Firmicutes, Proteobacteria, and Verrucomicrobia.

### Microbial diversity in the lungs of SARS-CoV-2-challenged K18-hACE2 mice.

**FIG 5:**
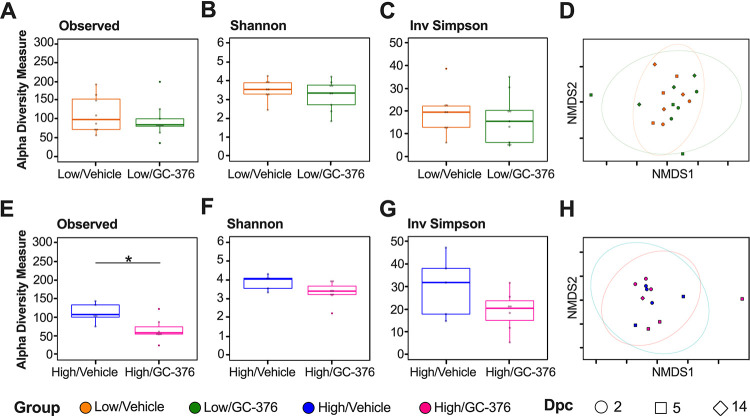
Alpha and beta diversity metrics of lung samples without/with antiviral GC-376. Comparison of observed ASVs (A), Shannon diversity index (B), and Inv Simpson (C) of the low-dose groups without (low/vehicle, orange) and with antiviral (low/GC-376, green) containing all time points (dpc) from rarified ASV count table. (D) NMDS plot of weighted Bray-Curtis dissimilarity distance of the rarified ASV count table of the low-dose groups. Days postchallenge are indicated by the shape and groups by color. Ellipses were constructed using a multivariate *t* distribution. Comparison of observed ASVs (E), Shannon diversity index (F), and Inv Simpson (G) of the high-dose groups without (high/vehicle, blue) and with antiviral (high/GC-376, pink). (H) NMDS plot of weighted Bray-Curtis dissimilarity distance of the rarified ASV count table of the high-dose groups. Days postchallenge are indicated by the shape and groups by color. Ellipses were constructed using a multivariate *t* distribution. All statistical tests were performed using Kruskal-Wallis or Wilcox-rank test for pairwise comparisons. *, *P* < 0.05.

Since we observed intestinal dysbiosis in SARS-CoV-2-infected mice, we subsequently analyzed the changes of the antiviral M^pro^ inhibitor GC-376 and infection in the lung microbiome. The effect of GC-376 was analyzed from homogenized lung tissue samples, and we specifically investigated how antiviral treatment may have affected microbiome changes in the lung during SARS-CoV-2 infection. Due to low DNA concentrations and <10,000 reads/sample, we did not include the six lung samples from the PBS/vehicle group in our analyses. Therefore, analysis of the microbiome was only assessed in lung samples from low/vehicle, high/vehicle, low/GC-376, and high/GC-376 groups. The low-virus-dose challenge groups showed no significant differences among the numbers of observed ASVs and the Shannon or Inv Simpson diversity indexes between GC-376-treated and untreated mice when samples from all time points were considered (Fig. 5A, B, and C). Bray-Curtis dissimilarity distances similarly did not show treatment-specific differences (Fig. 5D) (PERMANOVA *P* = 0.88). Results from the two high-virus-dose challenge groups showed that the high/vehicle group had a significantly higher combined number of ASVs than the high/GC-376 group (Fig. 5E). However, the Shannon and Inv Simpson diversity indexes were similar (Fig. 5F and G), and the Bray-Curtis dissimilarity did not cluster by treatment group or show treatment-specific differences (Fig. 5H) (PERMANOVA *P* = 0.72). Collectively, the results showed that the lung microbial diversity during infection with SARS-CoV-2 was unaffected by the antiviral treatment at low or high virus challenge doses.

**FIG 6:**
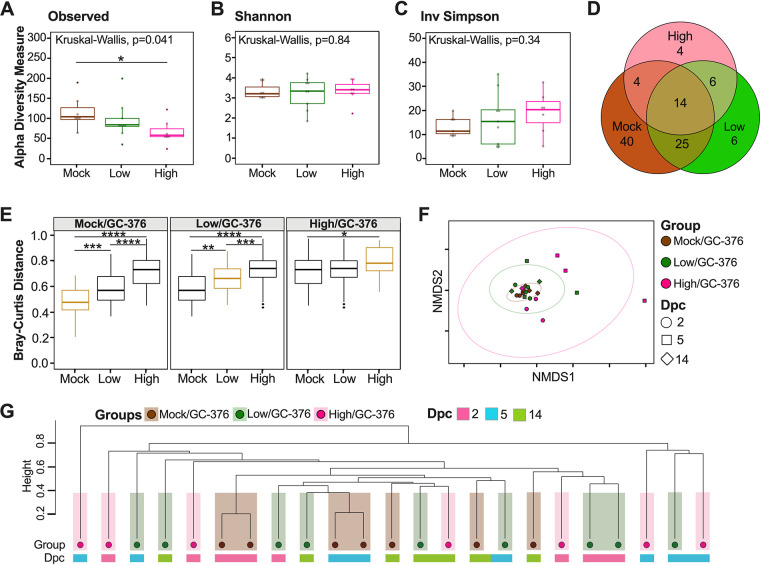
Alpha and beta diversity metrics of lung samples. Comparison of observed ASVs (A), Shannon diversity index (B), and Inv Simpson (C) of different groups (mock/GC-376, brown; low/GC-376, green; high/GC-376, pink) containing all time points from rarified ASV count table. (D) Venn diagram of rarified counts comparing the three different groups. (E) Comparison of Bray-Curtis dissimilarity distances within each group and across different groups. Gold boxes represent within variation while black boxes represent other groups. (F) NMDS plot of weighted Bray-Curtis dissimilarity distance of the rarified ASV count table. Days postchallenge are indicated by the shape and group by color. Ellipses were constructed using a multivariate *t* distribution. (G) Dendrogram showing the relationships of different groups and time points (dpc) using Bray-Curtis dissimilarity distance. Hierarchical cluster analysis was performed using hclust with agglomeration method average. Shaded colors and circles correspond to the different groups described previously. Colored bars below the circles represent the different time points (pink, 2 dpc; light blue, 5 dpc; green, 14 dpc). All statistical tests were performed using Kruskal-Wallis or Wilcox-rank test for pairwise comparisons. *, *P* < 0.05; **, *P* < 0.005; ***, *P* < 0.0005; ****, *P* < 0.00005.

Because the PBS/vehicle treatment did not yield high-quality data and similar lung microbial diversity was observed regardless of whether antiviral treatment took place, alpha and beta diversity metrics for the lung microbiome were compared for the GC-376-treated groups (mock/GC-376, low/GC-376, and high/GC-376) to examine the impact of infection on the lung microbiome. The numbers of observed ASVs (Kruskal-Wallis *P* = 0.041) (Fig. 6A) varied significantly among groups. The mock/GC-376 group had the highest number of ASVs, followed by low/GC-376 and then high/GC-376; the mock/GC-376 group had significantly greater number of ASVs compared to high/GC-376 groups, including all time points (Wilcox-rank test *P* = 0.021) (Fig. 6A). Analyses at different days postchallenge revealed similar numbers of ASVs regardless of time point for the the mock/GC-376 group, whereas virus challenge groups had decreased numbers of ASVs as the infection progressed, aligning with a large decrease in body weight change (see Fig. S3). In contrast to that in the ceca, Shannon and Inv Simpson indexes for the microbiome in lungs of the GC-376-treated groups were not significantly different when comparing across viral doses or over the course of infection (Fig. 6B and C; Fig. S3 and S4).

Since the alpha diversity analysis suggest limited to no differences among SARS-CoV-2-infected mice in the lungs, we next analyzed the number of shared ASVs among the different groups (Fig. 6D). The three GC-376-treated groups shared 14 ASVs (rarified count data, detection limit of 0.001 in at least 90% of the samples) (Fig. 6D). Following the same criteria, the mock/GC-376 group had 40 unique ASVs, while the low/GC-376 and the high/GC-376 groups had 6 and 4 unique ASVs, respectively (Fig. 6D). The low/GC-376 group shared 25 ASVs with the mock/GC-376 group and 6 ASVs with the high/GC-376 group (Fig. 6D). To further understand the differences among groups, we quantified the change of the lung microbiome composition among different groups by comparing Bray-Curtis distances within and across groups (Fig. 6E). The results suggest that the high/GC-376 group was most dissimilar to the others, while the low/GC-376 group and the mock/GC-376 group were more similar (Fig. 6E). In addition, the high/GC-376 group showed greater within-group variation than low/GC-376 and mock/GC-376 groups (Fig. 6E). Next, an NMDS plot of the Bray-Curtis dissimilarity distance was used to assess the relationship between the lung microbial community and SARS-CoV-2 challenge during the course of infection (Fig. 6F). Bray-Curtis dissimilarity NMDS showed tight grouping of samples with outliers that belonged to the high/GC-376 group at 5 dpc, which was supported by a PERMANOVA analyzing the difference among groups (*P* = 0.01) (Fig. 6F). Furthermore, we investigated the relationship of the three groups across days postchallenge by producing a hierarchical cluster analysis using the Bray-Curtis dissimilarity distances (Fig. 6G). The lung microbiota showed less clustering by treatment than that of the ceca (Fig. 6G). Altogether, the results suggest that there are dose-dependent changes in microbial community composition following SARS-CoV-2 infection, although the results are not significant, unlike those for the cecal microbiome.

**FIG 7:**
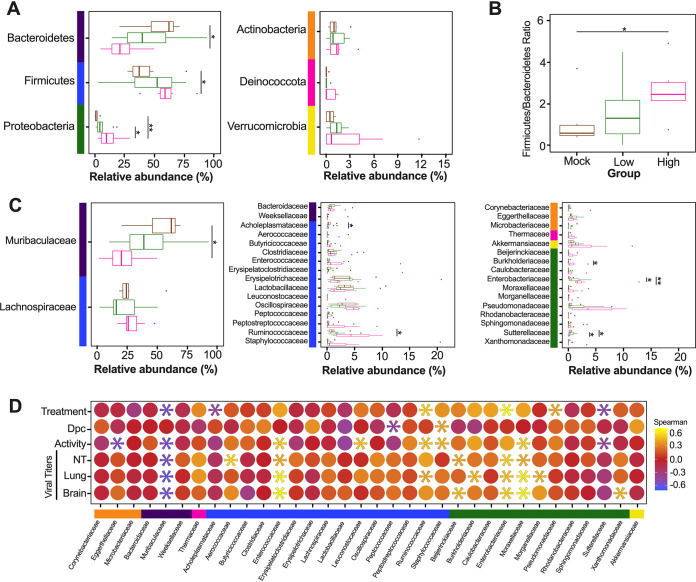
Relative abundance (%) of microbial communities in the lung at the phylum and family levels. (A) Relative abundances (%) of the most abundant phyla were compared via box plots for each group (mock/GC-376, brown; low/GC-376, green; high/GC-376, pink). Each box represents the interquartile range (first and third quartiles) of taxon abundance, and the line corresponds to the median abundance. Vertical lines represent variation in abundance, and the circles represent outliers. Corresponding phyla are noted by the colored bar on the left (purple, Bacteroidetes; blue, Firmicutes; green, Proteobacteria; orange, Actinobacteria; pink, Deinococcota; yellow, Verrucomicrobia). (B) Firmicutes/Bacteroidetes ratios were calculated and graphed to analyze differences among groups. One outlier for a sample from high/GC-376 group is not shown (F/B = 17). (C) Relative abundances (%) of the most abundant families were compared via box plots. Corresponding phyla are noted by the colored bar on the left and in accordance with that in panel A. One outlier each from low/GC-376 (Lactobacillaceae, 33.25%) and high/GC-376 (Staphylococcaceae, 39.8%) groups are not shown. (D) Spearman correlation by family abundance. Corresponding phyla are noted by the colored bar on the left and in accordance with that in panel A. Significant values (*P* < 0.05) are demonstrated as an asterisk, while nonsignificant correlations are shown as a circle. Correlative factors include treatment (administered PBS, a low dose, or high dose), days postchallenge (2, 5, or 14), activity score (response to the environment and personnel stimulation; higher score indicates decreased activity), or viral titers measured in the nasal turbinates (NT), lung, and brain. Data (activity score, viral titers in the NT, lung and brain) were adapted from Cáceres et al. (43). All statistical tests were performed using Wilcox-rank test for pairwise comparisons. *, *P* < 0.05; **, *P* < 0.005.

**FIG 8:**
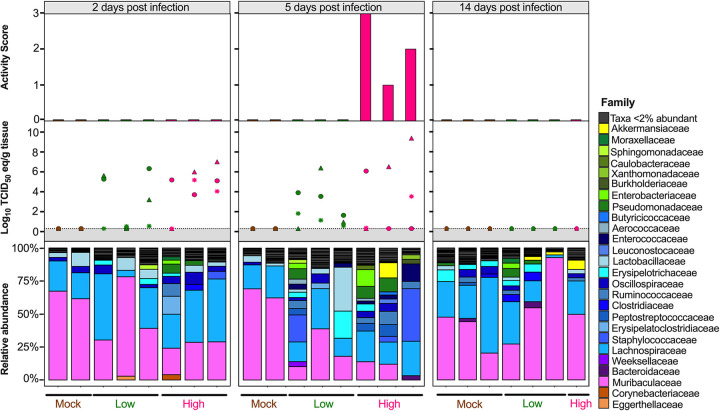
Relative abundance (%) of microbial communities in the lung at the family level. Relative abundances (%) of individuals were calculated by agglomerating at the family level and then transformed into relative abundances. Taxa that had less than 2% abundance were grouped together. Groups are indicated by the bars at the bottom and by color (mock/GC-376, brown; low/GC-376, green; high/GC-376, pink). Each set of graphs is separated by time point indicated at the top. Viral nasal turbinates (circle), lung (triangle), and brain (asterisk) titers of individual mice that underwent microbial analysis are shown in the box above the microbial taxonomic abundances. Activity scores (response to the environment and personnel stimulation; higher score indicates decreased activity) for each mouse are shown above the viral titers. Data including activity score, viral titers in the NT, lung and brain were adapted from Cáceres et al. 2021 (43).

Considering the diversity metrics suggested a limited difference in infected and control mice, we further analyzed the relative abundances of the microbial communities at the phylum and family levels in the lungs. The most abundant phyla within the lungs were Bacteroidetes, Firmicutes, Proteobacteria, Actinobacteria, Deinococcota (Deinococcus-Thermus), and Verrucomicrobia (Fig. 7A). In contrast to that in the ceca, Bacteroidetes were suppressed in GC-376-treated mice exposed to low- and high-dose virus, with the mock/GC-376 exhibiting significantly higher abundance of Bacteroidetes than the high/GC-376 group (*P* = 0.017) (Fig. 7A). The high/GC-376 group had a significantly higher abundance of Firmicutes than the mock/GC-376 group (Wilcox-rank test *P* = 0.038) (Fig. 7A). The low/GC-376 and high/GC-376 groups had a significantly greater abundance of Proteobacteria than the mock/GC-376 group (Wilcox-rank test *P* = 0.025 and *P* = 0.0087, respectively) (Fig. 7A). Similar abundances were observed for Actinobacteria, Deinococcus-Thermus, and Verrucomicrobia across all groups (Fig. 7A). Following this, we looked at the relationship among Firmicutes and Bacteroidetes by analyzing the F/B ratio. Contrary to that in the ceca, the mock/GC-376 group had the lowest F/B ratio, followed by low/GC-376 and then high/GC-376 groups (Fig. 7B). Pairwise comparisons showed that the mock/GC-376 group had a significantly lower F/B ratio than the high/GC-376 group (Wilcox-rank test *P* = 0.02) (Fig. 7B). Despite having similar if not lower ASV-level diversity, lung samples showed higher family-level diversity than the cecum (Fig. 7C). Across all days postchallenge, the mock/GC-376 group had a significantly higher abundance of Muribaculaceae than the high/GC-376 group, similar to that for the parent phylum, Bacteroidetes (Wilcox-rank test *P* = 0.01) (Fig. 7C). Within the Firmicutes phylum, the mock/GC-376 group had a significantly higher abundance of Acholeplasmataceae than the high/GC-376 group (*P* = 0.04), while the high/GC-376 group had a significantly higher abundance of Ruminococcaceae than the mock/GC-376 (*P* = 0.01) (Fig. 7C). Furthermore, the high/GC-376 group had significantly increased abundance of Burkholderiaceae and Enterobacteriaceae within the Proteobacteria phyla compared to that in the mock/GC-376 group (*P* = 0.03 and *P* = 0.002, respectively) (Fig. 7C). Meanwhile, mock/GC-376 and low/GC-376 groups had significantly increased abundance of Sutterellaceae compared to that in the high/GC-376 group (Fig. 7C). While performing a correlation analysis among bacterial families and viral infection factors, multiple families within Actinobacteria and Bacteroidetes were negatively correlated with the severity of viral infection, while families within Firmicutes and, more prominently, Proteobacteria were positively correlated (Fig. 7D). In particular, the family Enterobacteriaceae was notably enriched at 5 dpc in all individuals within high/GC-376 group (Fig. 8). Collectively, the taxonomic relative abundances displayed distinct changes at the phylum and family levels in mice challenged with low and high challenge doses of the SARS-CoV-2 virus and treated with GC-376, particularly in families within the phyla Bacteroidetes, Firmicutes, and Proteobacteria.

## DISCUSSION

We analyzed the cecum and lung microbiome changes that occur in K18-hACE2 mice upon challenge with two different doses of a prototypical SARS-CoV-2 virus. Some limitations of this study must be noted. While the environment was stable and controlled, the sample size for each group at each time point was small, and the potential contribution of cage effect on the microbiome was not analyzed. The high mortality observed in the high-virus-dose groups only allowed for collection of two time points (2 and 5 dpc, only one sample was collected at 14 dpc from the only survivor in the high/GC-376 group). As indicated above, cecum samples were only collected for mice not treated with GC-376. In contrast, lung samples from PBS/vehicle-treated control mice did not yield sufficient amplifiable microbial DNA for sequencing, and so the comparison focused on SARS-CoV-2 dose-dependent responses in GC-376-treated mice.

The microbiome of the cecum showed significant decreases in Shannon and Inv Simpson indexes comparing the control to the low-dose- and high-dose-infected groups (Fig. 2B and C). The low-virus-dose group shared a higher number of ASVs with the control group than with the high-virus-dose group (Fig. 2D). These observations suggest a virus dose-dependent effect of the ceca microbial alpha diversity in mice infected with SARS-CoV-2. While preparing the manuscript, a report was published that analyzed the small intestine microbiome of hACE2 mice among unvaccinated and vaccinated mice challenged with a high dose of SARS-CoV-2 (47). While we compared control mice to nonvaccinated mice challenged with low and high doses of SARS-CoV-2 in this study, findings were similar, since a decrease in alpha diversity in the unvaccinated mice was reported, which was consistent with results obtained from human fecal samples (5, 47). From the beta diversity analysis, the weighted Bray-Curtis NMDS showed that samples from the high-virus-dose group were outliers compared to the rest of the samples, consistent with exacerbation of clinical signs and following peak virus replication in this group (40, 43, 44, 48). Further analysis of the Bray-Curtis distances of cecal samples showed that the low-dose group was more similar to the control group than to the high-dose group (Fig. 2F and G), further suggesting that microbial change is virus dose dependent. A similar relationship was previously observed among unvaccinated and vaccinated SARS-CoV-2-infected mice with a high dose (47). Analyzing the commensal microbiome in other diseases has shown that the microbiota can both regulate and be regulated by viral pathogens and facilitate stimulatory or suppressive effects on the host immune response (49). It is possible that the distinct clustering and change observed at 5 dpc in the cecal microbiome of mice infected with a high virus dose corresponding to increased clinical signs and weight loss (see Fig. S1 in the supplemental material and Fig. 4) could be caused by a hyperactive host innate immune response and/or SARS-CoV-2 virus replication. It is also possible that cecal microbiome changes could contribute to the rapid increase of disease severity. Viral pathogens that infect or replicate in mucosal tissues most likely encounter commensal microbiota inhabiting the mucosal surfaces (50). Therefore, the intestinal microbiota can either promote viral infections, such as with poliovirus, reovirus, and certain retroviruses, or have a protective role, such as with influenza virus and rotavirus (51–54).

The most distinct differences in taxonomic relative abundance within the ceca of infected mice were the overall lower abundance of Firmicutes, particularly the families Erysipelotrichaceae, Lachnospiraceae, Lactobacillaceae, and Oscillospiraceae, the increased abundance of Proteobacteria in the low-virus-dose group, and the decreased abundance of Actinobacteria and increased abundance of Verrucomicrobia, particularly the family Akkermansiaceae, in the high-virus-dose group at 5 dpc (Fig. 4). In addition, a significant difference in the F/B ratios was observed among the control and high-dose groups (Fig. 3B). The F/B ratio has been associated with maintaining homeostasis, and changes could be indicative of dysbiosis (55). The results in this report showed a decrease in the F/B ratio within the ceca, which was also observed in patients with inflammatory bowel disease and in mice infected with RSV (20, 55). Multiple studies have reported a decrease in Firmicutes during respiratory viral infections in the intestinal microbiome, particularly in influenza virus infection (5, 18, 20). Interestingly, Firmicutes, particularly Lachnospiraceae, was not significantly decreased in SARS-CoV-2 stool samples compared to that in the controls in humans (5). Members of the Lachnospiraceae family are anaerobic fermentative bacteria that hydrolyze starches, sugars, and other short-chain fatty acids (SCFAs) (56). Previous reports have shown that SCFAs are important for the maintenance of colonic epithelial cells, directly interact with the host immune response, and promote bactericidal activity of alveolar macrophages during influenza virus infection (21, 56). As observed in this study, the decrease in Firmicutes, particularly Lachnospiraceae, Erysipelotrichaceae, and Lactobacillaceae, correlates with virus challenge dose (Fig. 3). Therefore, variations in analyses of human samples could be dependent on sample type and viral load during infection, as shown within this study. Previous studies have shown increased abundance of Proteobacteria during influenza virus infection in mice, similar to the results in this study (19, 57). The increase in Proteobacteria has been hypothesized to be mediated by type 1 interferons, which have been shown to be impaired in severe SARS-CoV-2 cases but not in patients with mild-moderate outcomes (58, 59). The decrease in type 1 interferon in severe patients could potentially correlate with the significant increase in Proteobacteria with the low-virus dose compared to that with the high-virus dose found within this study; however, further research is needed to investigate this relationship. Finally, increases in abundance of the family Akkermansiaceae is of particular interest. The family Akkermansiaceae was classified further into one genus *Akkermansia*. A previous report showed that hACE2 mice that were not vaccinated and challenged with SARS-CoV-2 had a significantly increased abundance of *Akkermansia* compared to that in vaccinated challenged mice, similar to what was observed in this study among controls and the high-challenge-dose group (47). One of the primary *Akkermansia* species in the ceca of mice, *Akkermansia muciniphila*, a Gram-negative obligate anaerobe, has been shown to alter mucosal gene expression toward increased expression of genes involved in the immune response, particularly genes involved in antigen presentation of leukocytes (60, 61). We previously reported that mice from the high-virus-dose group had increased staining of CD3 and Iba-1, markers for cellular infiltration, and more pronounced neutrophilic inflammation than in the low-virus-dose and control groups (43). While 2 of the 3 high-virus-dose mice at 5 dpc were outliers compared to the other samples, one mouse was closer in proximity to the low-virus-dose and control groups in the Bray-Curtis NMDS and had a more similar taxonomic composition to these groups (Fig. 2F and G and Fig. 4). Looking closer at the taxonomic differences, the sample from this mouse also had lower relative abundance of *Akkermansia* than the other two mice at 5 dpc (Fig. 4). While challenged with the same dose, this mouse showed reduced clinical signs throughout infection, thus suggesting that the decreased abundance of *Akkermansia* in this mouse could be correlated to disease severity. A recent report showed that while the abundance of *Akkermansia* positively correlated with influenza H7N9 infection in mice, oral administration of *A. muciniphila* significantly reduced weight loss, mortality, and viral titers (22). Therefore, further research is needed to better understand the role of *Akkermansia* in severe SARS-CoV-2 infection. In conclusion, the cecal data suggest that the distinct changes of the cecum microbiome could be virus dose dependent, and specific taxa could play a role in the modulation of the immune response, potentially leading to multisystemic inflammatory syndrome, a major complication of SARS-CoV-2 infection (2–9).

While the intestinal microbiome has been the center of previous microbiome research, recently, multiple groups have analyzed the microbiome composition of the upper and lower respiratory tracts (16, 18, 62, 63). In particular, the lung microbiota is understood to provide resistance to the colonization of respiratory pathogens and immune tolerance (63). To our knowledge, no studies have analyzed the effects of an antiviral on the lung microbiome. We found no significant differences among groups of mice challenged with SARS-CoV-2 that were either treated or not treated with the M^pro^ inhibitor GC-376 (Fig. 5). Since GC-376 had limited effect on the clinical outcome of SARS-CoV-2 in mice, the limited differences in the microbial compositions in the lungs between treated and nontreated mice is not entirely surprising. Among the samples obtained from GC-376-treated mice and in contrast to samples from ceca, differences in alpha diversity indexes between control, low-dose-, and high-dose-infected mice were not observed besides decreased numbers of ASVs within the lungs (Fig. 6A). Similar to results within this study, previous reports also showed no significant differences in Shannon diversity within the lung microbiome of mice infected with influenza virus (18, 28). However, a recent study analyzing the nasopharyngeal microbiome of human SARS-CoV-2-positive patients showed diversity changes that correlated with disease severity (27). A potential explanation for differences among studies is sample type (nasopharyngeal compared to lungs), host species (humans compared to mice), or mitigated disease caused by GC-376 treatment. Regarding beta diversity analysis, the weighted Bray-Curtis NMDS did not show separation of clusters by treatment but rather a single overlapping cluster with outliers that primarily belong to the high-virus-dose group (Fig. 6F). Similarly, an analysis using influenza virus detected no significant changes in beta diversity of the lower respiratory tract throughout infection (18). However, analysis of Bray-Curtis dissimilarity indicated that the low-dose infected mice were more similar to the control group than to the high-dose infected mice (Fig. 6E and G) and that within-group variance increased in an infectious-dose-dependent manner, consistent with the Anna-Karenina model of disease-induced dysbiosis (64, 65). Comprehensively, the beta diversity analysis suggests that there are limited lung microbial composition changes, dissimilar to that in the ceca.

The most distinct differences in taxonomic relative abundance within the lungs of infected mice are the overall lower abundance of Bacteroidetes, higher abundance of Firmicutes, and higher abundance of Proteobacteria in the low- and high-virus-dose groups than in the mock control (Fig. 6), which is consistent with previous reports (66, 67). Firmicutes and Proteobacteria were enriched in the high-and low-virus-dose mice compared to that in the mock controls, consistent with previous reports of patients infected with influenza virus (68). A significant difference among the F/B ratios was observed between the control and high-dose groups, suggesting dysbiosis in the lung microbiome post-SARS-CoV-2 infection with a high dose. Similar results were observed in patients that underwent lung transplants, but to our knowledge, this has not been thoroughly examined in respiratory viral infections (69).

Future studies are needed with larger group sizes, cage effect compensation, and analysis of different sections of the intestinal tract (duodenum, jejunum, and ileum) and respiratory tract (lower and upper sections) to better understand the role of microbiome changes during SARS-CoV-2 infection. However, the proof-of-principle approach of this report identified significant changes in the cecal and lung microbiomes of K18-hACE2 mice, particularly those challenged with a high dose of the SARS-CoV-2 virus, that warrant more in-depth studies.

## MATERIALS AND METHODS

### Ethics statement.

Animal studies were approved by the Institutional Animal Care and Use Committee (IACUC) of the University of Georgia (protocol A2019-03-032-Y1-A3) and performed according to the IACUC guidebook of the Office of Laboratory Animal Welfare and Public Health Service (PHS) policy on humane care and of use of laboratory animals. Animals were humanely euthanized according to guidelines by the American Veterinary Medical Association (AVMA). Studies were performed in an animal biosafety level 3 containment facility at the Animal Health Research Center (AHRC) at the University of Georgia.

### Cells and virus.

The SARS-CoV-2 isolate (isolate USA-WA1/2020), kindly provided by S. Mark Tompkins, Department of Infectious Diseases, University of Georgia, was used for virus challenge in the animal studies. Virus propagation and titration are explained in detail in the study by Caceres et al. (43). Briefly, the virus was grown in Vero E6 Pasteur cells provided by Maria Pinto (Center for Virus Research, University of Glasgow, Scotland, UK) and maintained in Dulbecco’s modified Eagles medium (DMEM, Sigma-Aldrich, St. Louis, MO) containing 10% fetal bovine serum (FBS; Sigma-Aldrich), 1% antibiotic-antimycotic (AB; Sigma-Aldrich), and 1% l-glutamine (Sigma-Aldrich). Cells were cultured at 37°C under 5% CO_2_ for 96 h. Virus stocks were titrated by 50% tissue culture infectious dose (TCID_50_), and virus titers were established according to the Reed and Muench method (70).

### Mouse experiments.

Female K18-hACE2 mice (6 weeks old) were randomly distributed into six groups (*n* = 6/group for controls and *n* = 9/group for challenged), anesthetized, and challenged intranasally with 50 μl of phosphate-buffered saline (PBS), 1 × 10^3^ TCID_50_/mouse (low virus dose) or 1 × 10^5^ TCID_50_/mouse (high virus dose). At 3 h postchallenge, GC-376 (20 mg/kg body weight/dose, 40 mg/kg body weight daily), kindly provided by Jun Wang (Department of Pharmacology and Toxicology, University of Arizona), or vehicle (H_2_O) was administered to each mouse via intraperitoneal (i.p.) injection twice per day and continued for 7 days (Fig. 1A). Mice were monitored twice a day for clinical signs of disease postchallenge. Mice were humanely euthanized if they lost ≥25% of their initial body weight (a score of 3 on a 3-point scale of disease severity). At 2 and 5 dpc, a subset of mice was humanely euthanized, *n* = 2/time point from PBS/vehicle and mock/GC-376 groups and *n* = 3/time point for the low (low/vehicle and low/GC-376) and high (high/vehicle and high/GC-376) doses. Cecum and lungs from each mouse were collected and stored at −80°C until further analysis. At 14 dpc, the same procedure was performed with all of the remaining animals (PBS/vehicle, mock/GC-376, low/vehicle, low/GC-376, and high/GC-376 groups) (Fig. 1A).

### Tissue sample preparation.

Tissue homogenates were generated using the TissueLyser II (Qiagen, Gaithersburg, MD). In summary, 500 μl of PBS-AB was added to each sample (lungs, 0.01 to 0.04 g; cecum, 0.3 to 0.5 g) along with tungsten carbide 3-mm beads (Qiagen). Samples were homogenized at a speed of 10 Hz for 10 min. Homogenized tissue was stored at −80°C until further analysis.

### DNA extraction, amplicon library preparation, and sequencing.

DNA was extracted from the tissue homogenates using an MoBio Power Soil kit (Qiagen, Gaithersburg, MD) with minor changes according to the earth microbiome protocol as follows: additional incubation at 65°C for 10 min after the addition of solution C1, beads were shaken at 20 Hz for 20 min instead of 10 min, and samples were incubated at 4°C for 10 min instead of 5 min and then stored at −80°C until use. Following extraction, the microbial 16S rRNA gene was amplified using Phusion Hot Start 2 DNA polymerase (Thermo Fisher, Waltham, MA) and V4 hypervariable region of the 16S rRNA gene primers 515F (5′-GTGCCAGCMGCCGCGGTAA-3′) and 806R (5′-GGACTACHVGGGTWTCTAAT-3′) in 20-μl PCRs (8.9 μl of molecular-grade water, 4 μl of 5× HF buffer, 0.4 μl of 10 mM deoxynucleoside triphosphates [dNTPs], 1.25 μl of 10 μM 515F, 1.25 μl of 10 μM 806R, 4 μl of DNA, and 0.2 μl of polymerase) under the following conditions: 98°C (30 s), followed by 25 cycles of 98°C (10 s), 52°C (30 s), and 72°C (30 s), a final elongation step at 72°C (5 min), and held at 4°C. The PCRs were performed in duplicates, and products were visualized on a 1% agarose gel. Duplicate PCR products of the same sample were pooled in equal volumes, cleaned by 0.45× of Agencourt AMPure XP magnetic beads (Beckman Coulter, Pasadena, CA) according to the manufacturer’s protocol, and eluted in molecular biology-grade water (Genesee Scientific, San Diego, CA). Amplicon concentration was measured using the Qubit dsDNA HS assay kit (Thermo Fisher) on a Qubit 3.0 fluorometer (Thermo Fisher). DNA concentrations were normalized to 1.0 ng/μl. Subsequently, amplified DNA was used in a secondary amplification/dual barcode annealing reaction. Forward and reverse dual barcode primers (primers and barcodes with different reference indexes) were designed based upon primers generated by Caporaso et al. (71). Secondary amplification reactions were performed using NEBNext high-fidelity 2× PCR master mix (NEB) in 50-μl reactions (26 μl of 2× mix, 20.5 μl of water, 1 μl of barcoded forward and reverse primers [10 μM], 1 μl of DNA) under the following conditions: 98°C (30 s), followed by four cycles of 98°C (10 s), 52°C (10 s), and 72°C (10 s), followed by six cycles of 98°C (10 s) and 72°C (1 min), followed by a final extension of 72°C (2 min) and then held at 4°C. Samples were subsequently cleaned by 0.45× of Agencourt AMPure XP magnetic beads according to the manufacturer’s protocol and eluted in molecular biology-grade water. Fragment size distribution was analyzed on a subset of samples using the Agilent Bioanalyzer 2100 DNA-HS assay (Agilent, Santa Clara, CA, USA). Sample libraries were then normalized and pooled to a concentration of 2 or 0.5 nM based on a predicted total product size of ∼420 bp using the Qubit dsDNA HS assay kit on the Qubit 3.0 fluorometer. The loading concentration of the pooled libraries was 10 pM. Libraries were sequenced using Illumina MiSeq V2 chemistry (Illumina, San Diego, CA), 2 by 250 paired end. Negative controls, including an extraction blank and a PCR blank, were included in each sequencing run (2 runs total). Due to limited DNA concentrations, we were unable to sequence 5 of the 6 PBS/vehicle lung samples.

### Sequence processing and analysis.

Primer removal and demultiplexing was performed using Illumina BaseSpace using default settings. Sequence analysis was performed in R (72) with open-source software package DADA2 (version 1.16.0) (73). Each sequencing batch was processed separately until chimera removal. For each batch, the quality of the raw paired-end reads was visualized and used to determine appropriate truncation of read 1 (R1) by 10 bp and read 2 (R2) by 50 bp. After truncation, reads were discarded if they contained more than 2 maxEE “expected errors” or a quality score of less than or equal to 2. Following, each quality-filtered and trimmed read was processed independently by applying the trained DADA2 algorithm. The reads were then merged with a minimum overlap of 20 bp. After merging, both sequencing batches that were previously processed separately were combined, and chimeras were removed using the consensus method with default settings. Taxonomy was assigned in DADA2 using the native implementation of the naive Bayesian classifier using Silva v.38 database. A count table and taxonomy file were created and used for downstream analysis.

Prior to diversity analysis, potential sequence contaminants were identified using package decontam (version 1.8.0) (74) in RStudio (version 1.2.5042) (75). Briefly, potential contaminants were identified by using the prevalence-based contaminant identification, which relies on the principle that sequences from contaminating taxa have a higher prevalence in negative-control samples (extraction and PCR blanks) than in true samples (74). A threshold of 0.1 was used to identify contaminants. In total, 14 potential contaminants were identified by package decontam (see Table S1 in the supplemental material); however, all contaminants had biological relevance to the sample types collected except for one, *Gemmobacter*, which was removed from the data set. Following, reads that did not identify as Bacteria, contained uncharacterized phylum, or identified as chloroplast and/or mitochondria were removed using the phyloseq package (version 1.32) (76). Subsequently, two samples with less than 10,000 reads/sample were removed (lungs, low/vehicle group at 2 dpc and high/vehicle group at 5 dpc), and one sample (lungs, mock/GC-376 group at 14 dpc), considered an outlier according to Grubbs test on taxonomic abundance using the outlier package (*P* = 6.022e−07) (77), was removed.

Alpha diversity metrics, including observed number of amplicon sequence variants (ASVs) and Shannon diversity and inverse Simpson (Inv Simpson) indexes were calculated using phyloseq. Briefly, samples were rarified to 12,000 using command rrarefy using the vegan package (version 2.57) (78). Following, the rarified counts were imported into phyloseq, and diversity indexes were calculated using command estimaterichness. Results were graphed using ggplot2 (version 3.3.2) (79) and ggpubr package (version 0.4) (80). Statistical pairwise comparison employing the Wilcox rank test was performed across groups and time points (dpc). Viral titers and percent body weight loss data were adapted from Caceres et al. (43) and then graphed in Prism (v 9.1.0). The final plots were combined using Adobe Illustrator (version 25.0.1). A Venn diagram of unique and shared ASVs was created using the package microbiome (version 1.10) (81). Rarified count data were converted to relative abundances, and then ASVs that were common among groups were combined. ASVs with a limited detection of 0.001 in at least 90% of the samples were included. The Venn diagram was graphed using package eulerr (version 6.1.0) (82). Regarding beta diversity, a weighted Bray-Curtis dissimilarity matrix was calculated with a minimum of 20 and maximum of 100 random starts using the rarified count data in vegan. A nonmetric multidimensional scaling (NMDS) plot was used to graph the dissimilarity matrix using ggplot2. Ellipses were constructed using command stat_ellipse in ggplot2 with a multivariate *t* distribution. All distances displayed in boxplots for comparison of within and across group Bray-Curtis dissimilarities were extracted from the same distance matrix as the one used for the NMDS and graphed using ggplot2. Hierarchal cluster analysis of the Bray-Curtis distances was created using command hclust with agglomeration method “average” (unweighted pair group method using average linkages [UPGMA]) producing a cophenetic correlation coefficient of 0.79. The dendrogram was created using the function plot, and shading/group colors were added using Adobe Illustrator (Version 25.0.1). Multivariate statistics analysis was performed using permutational multivariate analysis of variance (PERMANOVA) tests using Bray-Curtis dissimilarity distances with 1,000 permutations generated using vegan command adonis2. A *P* value of <0.05 was considered significant. All other statistical tests were performed using Kruskal-Wallis or Wilcoxon signed-rank tests using package ggpubr.

Relative abundances at the phylum and family levels were generated using phyloseq. First, taxa were agglomerated at the phylum or family level and then transformed into relative abundance. The box plots of the relative abundances were generated using ggplot2. The three samples that were previously removed were not included in the analysis. Only phyla and families that had greater than 1% relative abundance in a group are shown in the boxplots. A rough estimation of Firmicutes/Bacteroidetes ratio was calculated by dividing the relative abundance of the reads assigned to Firmicutes by the relative abundance of the reads assigned to Bacteroidetes. Statistical pairwise comparison among groups was performed using Wilcoxon signed-rank test. A *P* value of <0.05 was considered significant. To better understand the relationship among families and different viral infection factors, a correlation analysis was performed. Spearman correlation analysis was calculated using the function rcorr from the package Hmisc at the family level (83). Only families that were analyzed in the boxplots (greater than 1% abundance in at least 1 sample and present in more than 1 sample) were included. *P* values of less than 0.05 were considered significant and visualized in a plot using ggplot2. Plots were edited in Adobe Illustrator. For the taxonomic bar plots, taxa that had less than 1% (ceca) or 2% (lung) abundance across all samples (separated by ceca and lungs) were grouped together. The box plots and bar plots of the relative abundances were generated using ggplot2. Viral titers and activity scores were adapted from Caceres et al. (43) graphed in Prism (v 9.1.0). The final plots were combined using Adobe Illustrator (version 25.0.1). Scripts used for analysis can be found on github at https://github.com/brittanyaseibert/Seibertetal_SARS_K18hACE2Mice.

### Data availability.

The 16S sequencing data set was deposited under BioProject PRJNA722991.

## Supplementary Material

Reviewer comments
