# Supplementary material for: Mild and Severe SARS-CoV-2 Infection Induces Respiratory and Intestinal Microbiome Changes in the K18-hACE2 Transgenic Mouse Model
Source: Microbiol Spectr. 2021 Aug 11;9(1):10.1128/spectrum.00536-21. doi: 10.1128/spectrum.00536-21 (PMC8455067; doi:10.1128/spectrum.00536-21)
Supplement: SUPPLEMENTAL FILE 1 — Supplemental material. Download SPECTRUM00536-21_Supp_1_seq11.pdf, PDF file, 0.9 MB [file spectrum00536-21_supp_1_seq11.pdf]

**SUPPLEMENTAL FIGURES**

**Figure 1S. Alpha diversity metrics of ceca across time**. **(A)** Comparison of Observed ASVs, **(B)** Shannon diversity index and **(C)** Inverse Simpson of different groups (PBS/Vehicle Black, Low/Vehicle: Orange, High/Vehicle: Blue) separated by dpc from rarified ASV count table. Since the sample size was limited (n=2 or 3), pair-wise comparisons were not performed. Viral nasal turbinates (circle), lung (triangle) and brain (asterisk) titers of each group among their respective dpc are shown above the alpha diversity measure. Percent body weight change for each group at the respective dpc (2, 5, and 12) is shown above the viral titers. Data including body weight change and viral titers in the NT, lung and brain was adapted from Cáceres et al. 2021 (43).

**Figure 2S. Alpha diversity metrics of cecum samples across time**. **(A)** Comparison of Observed ASVs, **(B)** Shannon diversity index and **(C)** Inverse Simpson of infected groups (Low/Vehicle and High/Vehicle) separated by dpc from rarified ASV count table. All statistical tests were performed using Kruskal-Wallis or Wilcox-rank test for pair-wise comparisons using * = p<0.05 ; ** = p<0.005; *** = p<0.0005; **** = p<0.00005.

**Figure 3S. Alpha diversity metrics of lung samples across time**. **(A)** Comparison of Observed ASVs, **(B)** Shannon diversity index and **(C)** Inverse Simpson of different groups (Mock/GC-376: Brown, Low/GC-376: Green, High/GC-376: Pink) separated by dpc from rarified ASV count table. Since the sample size was limited (n=2 or 3) pair-wise comparisons were not performed. Viral nasal turbinates (circle), lung (triangle) and brain (asterisk) titers of each group among their respective dpc are shown above the alpha diversity measure. Percent body weight change for each group at the respective dpc (2 , 5, and 12 dpc) is shown above the viral titers. Data including body weight change and viral titers in the NT, lung and brain was adapted from Cáceres et al. 2021 (43).

**Figure 4S. Alpha diversity metrics of lung samples across time**. **(A)** Comparison of Observed ASVs, **(B)** Shannon diversity index and **(C)** Inverse Simpson of infected groups (Low/GC-376 and High/GC-376) separated by dpc from rarified ASV count table. All statistical tests were performed using Kruskal-Wallis or Wilcox-rank test for pair-wise comparisons using * = p<0.05 ; ** = p<0.005; *** = p<0.0005; **** = p<0.00005.

**Figure 5S. Bray-Curtis Dissimilarity distances of lung samples without / with antiviral GC-376.** Comparison of Bray-Curtis dissimilarity distances of mice infected with a A) low dose or (B) high dose of SARS-CoV-2 with or without GC-376. Gold boxes represent within-group variation while black boxes represent the between-group variation.

**TABLE S1**

*Identification of Potential Contaminants by Decontam*

| ASV ID | Kingdom | Phylum | Class | Order | Family | Genus |
|---|---|---|---|---|---|---|
| ASV_249 | Bacteria | Firmicutes | Clostridia | Oscillospirales | Oscillospiraceae | Colidextribacter |
| ASV_314 | Bacteria | Bacteroidota | Bacteroidia | Flavobacteriales | Weeksellaceae | Empedobacter |
| ASV_355 | Bacteria | Firmicutes | Bacilli | Lactobacillales | Vagococcaceae | Vagococcus |
| ASV_395 | Bacteria | Proteobacteria | Gammaproteobacteria | Pseudomonadales | Moraxellaceae | Acinetobacter |
| ASV_398 | Bacteria | Proteobacteria | Gammaproteobacteria | Pseudomonadales | Moraxellaceae | Acinetobacter |
| ASV_470 | Bacteria | Proteobacteria | Alphaproteobacteria | Rhodobacterales | Rhodobacteraceae | Gemmobacter |
| ASV_522 | Bacteria | Firmicutes | Clostridia | Oscillospirales | Oscillospiraceae | NA |
| ASV_635 | Bacteria | Proteobacteria | Gammaproteobacteria | Pseudomonadales | Moraxellaceae | Acinetobacter |
| ASV_680 | Bacteria | Proteobacteria | Alphaproteobacteria | Sphingomonadales | Sphingomonadaceae | Sphingomonas |
| ASV_714 | Bacteria | Firmicutes | Bacilli | Lactobacillales | Streptococcaceae | Lactococcus |

| | | | | | | |
|---|---|---|---|---|---|---|
| ASV_725 | Bacteria | Proteobacteria | Gammaproteobacteria | Pseudomonadales | Pseudomonadaceae | Pseudomonas |
| ASV_739 | Bacteria | Proteobacteria | Gammaproteobacteria | Burkholderiales | Comamonadaceae | NA |
| ASV_761 | Bacteria | Firmicutes | Clostridia | Oscillospirales | Oscillospiraceae | Flavonifractor |
| ASV_865 | Bacteria | Firmicutes | Clostridia | Lachnospirales | Lachnospiraceae | NA |

33

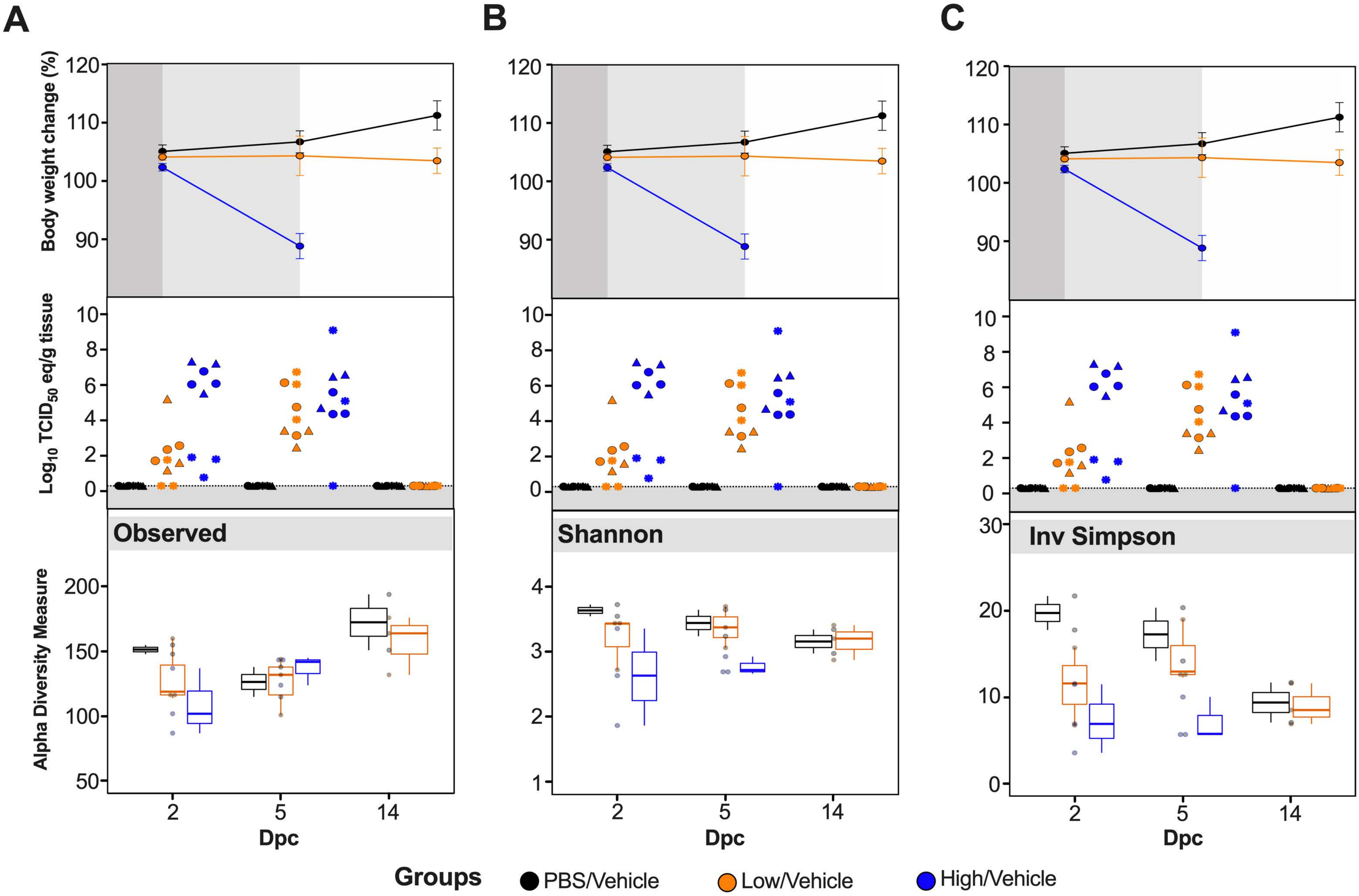

A
B
C
Body weight change (%)
120
110
100
90
Log10 TCID50 eq/g tissue
10
8
6
4
2
0
Observed
Shannon
Inv Simpson
Alpha Diversity Measure
200
150
100
50
4
3
2
1
30
20
10
0
2
5
14
Dpc
Groups
PBS/Vehicle
Low/Vehicle
High/Vehicle

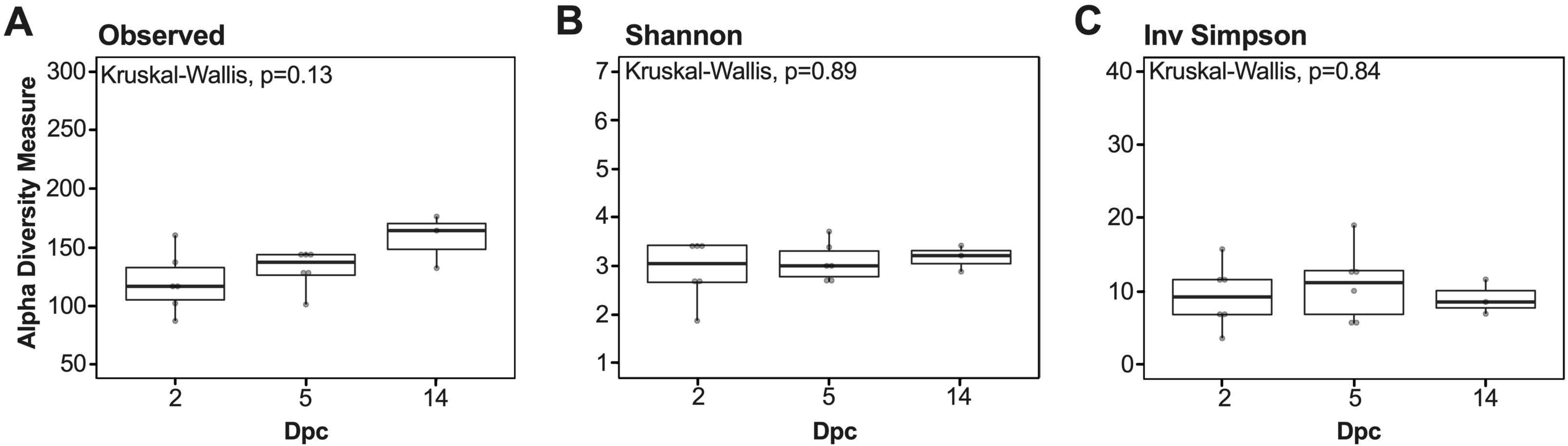
A
Observed
Kruskal-Wallis, p=0.13
Alpha Diversity Measure
300
250
200
150
100
50
2
5
14
Dpc
B
Shannon
Kruskal-Wallis, p=0.89
7
6
5
4
3
2
1
2
5
14
Dpc
C
Inv Simpson
Kruskal-Wallis, p=0.84
40
30
20
10
0
2
5
14
Dpc

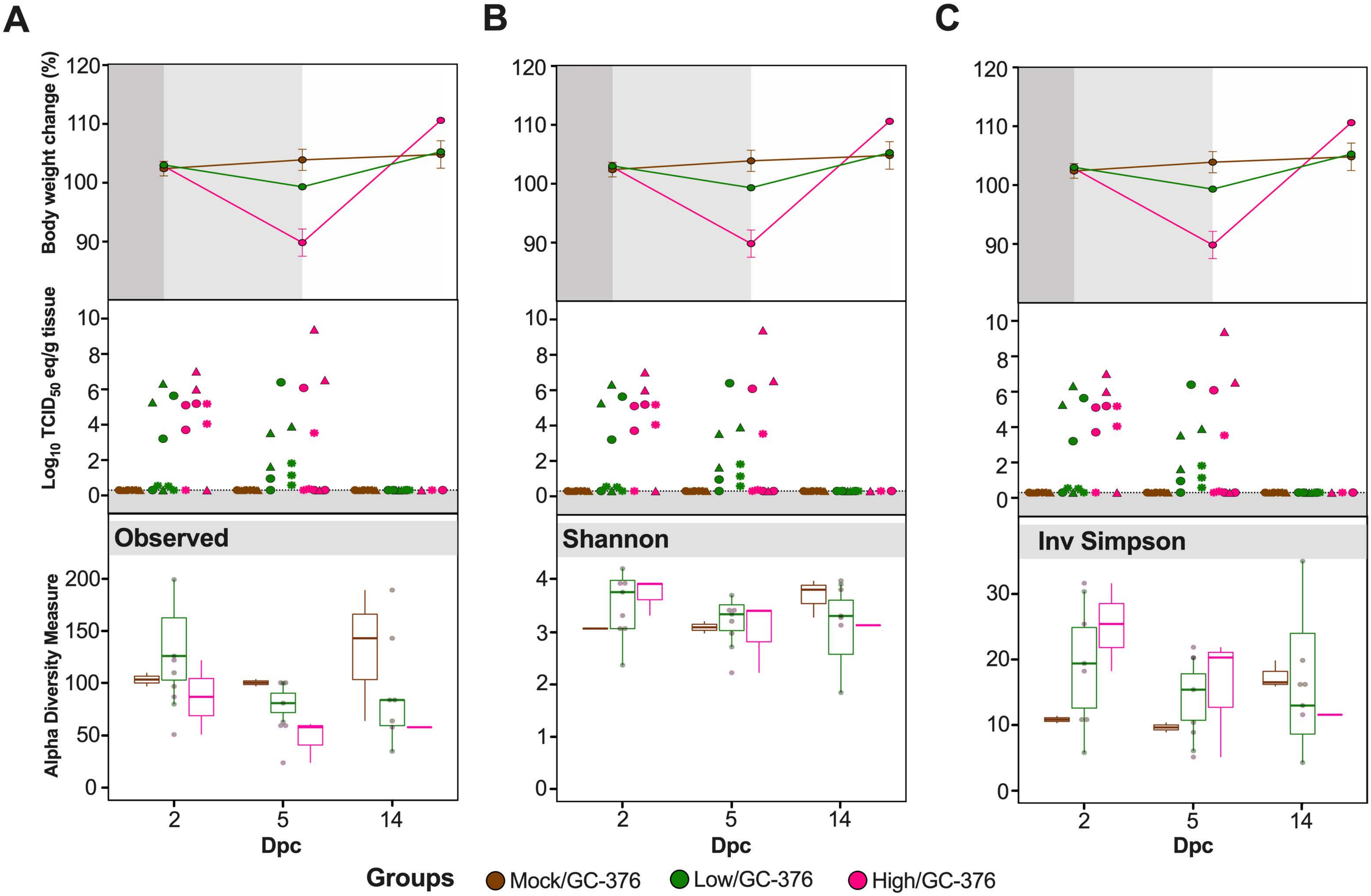

A
B
C
Body weight change (%)
120
110
100
90
Log10 TCID50 eq/g tissue
10
8
6
4
2
0
Observed
Shannon
Inv Simpson
Alpha Diversity Measure
200
150
100
50
0
4
3
2
1
30
20
10
2
5
14
Dpc
Groups
Mock/GC-376
Low/GC-376
High/GC-376

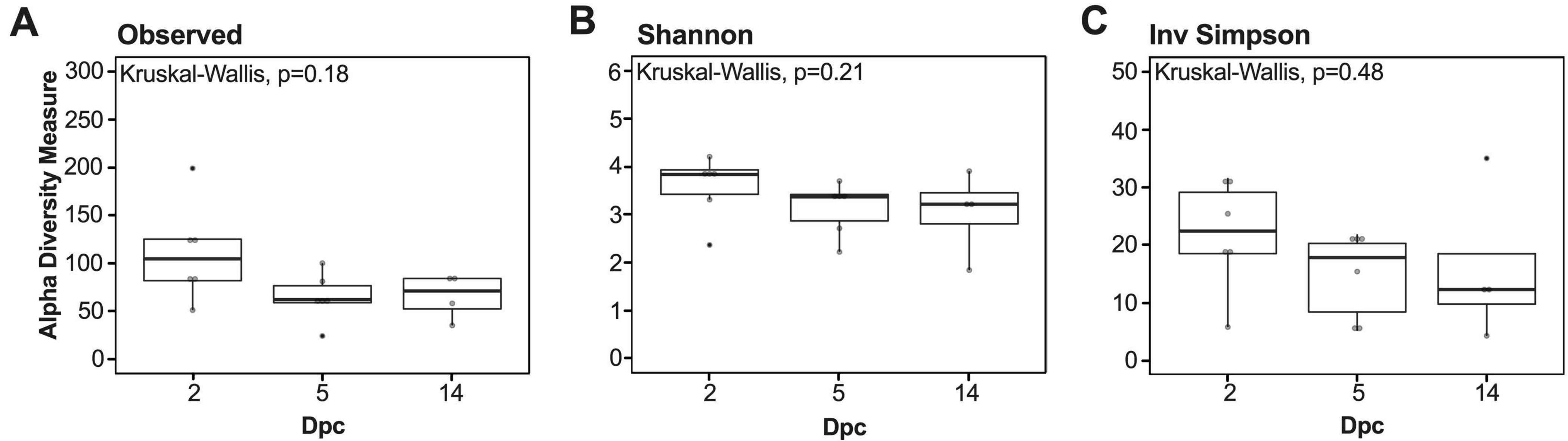

A
Observed
Kruskal-Wallis, p=0.18
Alpha Diversity Measure
300
250
200
150
100
50
0
2
5
14
Dpc
B
Shannon
Kruskal-Wallis, p=0.21
6
5
4
3
2
1
0
2
5
14
Dpc
C
Inv Simpson
Kruskal-Wallis, p=0.48
50
40
30
20
10
0
2
5
14
Dpc

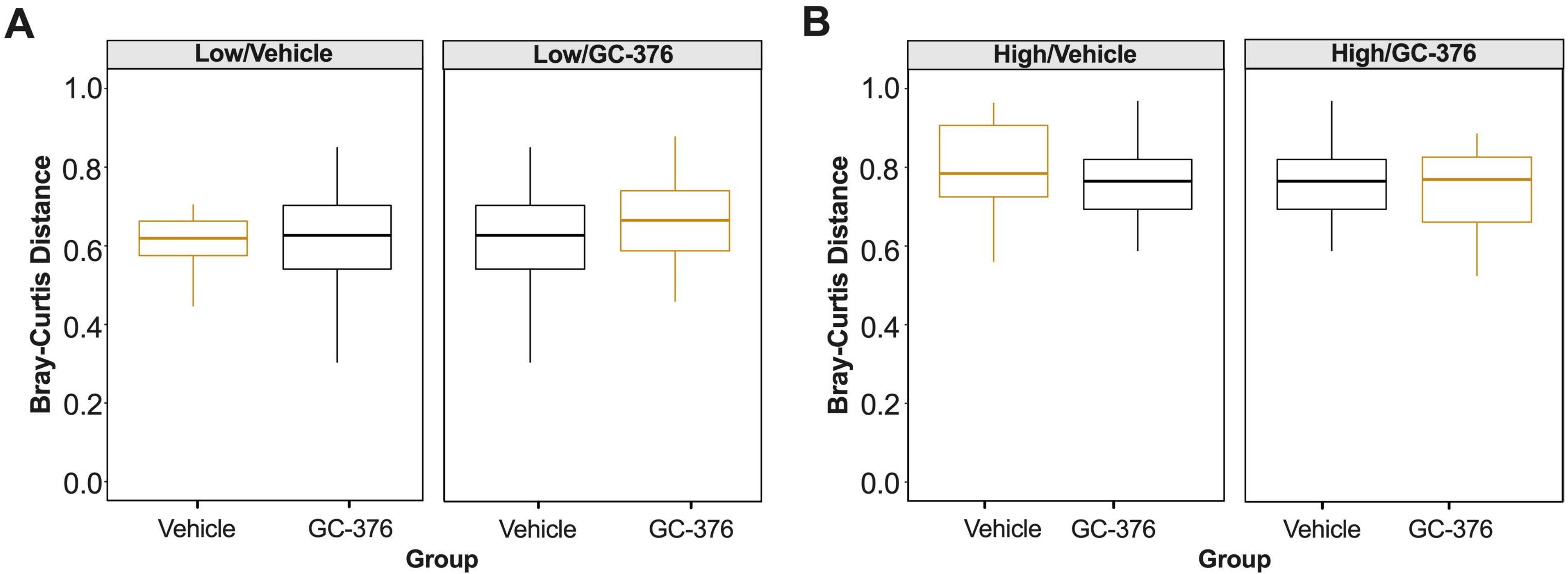
A
B
Low/Vehicle
Low/GC-376
High/Vehicle
High/GC-376
Bray-Curtis Distance
1.0
0.8
0.6
0.4
0.2
0.0
Vehicle
GC-376
Group
