# Supplementary material for: Mild and Severe SARS-CoV-2 Infection Induces Respiratory and Intestinal Microbiome Changes in the K18-hACE2 Transgenic Mouse Model
Source: Microbiol Spectr. 2021 Aug 11;9(1):10.1128/spectrum.00536-21. doi: 10.1128/spectrum.00536-21 (PMC8455067; doi:10.1128/spectrum.00536-21)
Supplement: Reviewer comments [file reviewer-comments.pdf]

AMERICAN SOCIETY FOR MICROBIOLOGY | Microbiology Spectrum

Mild and severe SARS-CoV-2 infection induces respiratory and intestinal microbiome changes in the K18-hACE2 transgenic mouse model

Brittany Seibert, C. Joaquin Caceres, Stivalis Cardenas-Garcia, Silvia Carnaccini, Ginger Geiger, Daniela Rajão, Elizabeth Ottesen, and Daniel Perez

Corresponding Author(s): Daniel Perez, University of Georgia

Review Timeline:

Submission Date:	June 18, 2021
Accepted:	July 15, 2021

Editor: Miguel Martinez

Reviewer(s): The reviewers have opted to remain anonymous.

Transaction Report:

(Note: With the exception of the correction of typographical or spelling errors that could be a source of ambiguity, letters and reports are not edited. The original formatting of letters and referee reports may not be reflected in this compilation.)

DOI: https://doi.org/10.1128/Spectrum.00536-21

July 15, 2021

Dr. Daniel R. Perez
University of Georgia
953 College Station Road
Athens, GA 30602

Re: Spectrum00536-21 (**Mild and severe SARS-CoV-2 infection induces respiratory and intestinal microbiome changes in the K18-hACE2 transgenic mouse model**)

Dear Dr. Daniel R. Perez:

Your manuscript has been accepted, and I am forwarding it to the ASM Journals Department for publication. You will be notified when your proofs are ready to be viewed.

The ASM Journals program strives for constant improvement in our submission and publication process. Please tell us how we can improve your experience by taking this quick Author Survey.

As an open-access publication, Spectrum receives no financial support from paid subscriptions and depends on authors' prompt payment of publication fees as soon as their articles are accepted. You will be contacted separately about payment when the proofs are issued; please follow the instructions in that e-mail. Arrangements for payment must be made before your article is published. For a complete list of **Publication Fees**, including supplemental material costs, please visit our website.

Corresponding authors may join or renew ASM membership to obtain discounts on publication fees. Need to upgrade your membership level? Please contact Customer Service at Service@asmusa.org.

Thank you for submitting your paper to Spectrum.

Sincerely,

Miguel Martinez
Editor, Microbiology Spectrum

Journals Department
American Society for Microbiology
1752 N St., NW
Washington, DC 20036
E-mail: spectrum@asmusa.org

Supplemental Material: Accept
